# Preparation and Characterization of Carboxymethyl Cellulose-Based Bioactive Composite Films Modified with Fungal Melanin and Carvacrol

**DOI:** 10.3390/polym13040499

**Published:** 2021-02-05

**Authors:** Łukasz Łopusiewicz, Paweł Kwiatkowski, Emilia Drozłowska, Paulina Trocer, Mateusz Kostek, Mariusz Śliwiński, Magdalena Polak-Śliwińska, Edward Kowalczyk, Monika Sienkiewicz

**Affiliations:** 1 Center of Bioimmobilisation and Innovative Packaging Materials, Faculty of Food Sciences and Fisheries, West Pomeranian University of Technology Szczecin, Janickiego 35, 71-270 Szczecin, Poland; emilia_drozlowska@zut.edu.pl (E.D.); p.trocer@gmail.com (P.T.); mkosa9406@gmail.com (M.K.); 2 Chair of Microbiology, Immunology and Laboratory Medicine, Department of Diagnostic Immunology, Pomeranian Medical University in Szczecin, Powstańców Wielkopolskich 72, 70-111 Szczecin, Poland; pawel.kwiatkowski@pum.edu.pl; 3 Dairy Industry Innovation Institute Ltd., Kormoranów 1, 11-700 Mrągowo, Poland; mariusz.sliwinski@iipm.pl; 4 Chair of Commodity Science and Food Analysis, Faculty of Food Science, University of Warmia and Mazury in Olsztyn, Pl. Cieszyński 1, 10-957 Olsztyn, Poland; m.polak@uwm.edu.pl; 5 Department of Pharmacology and Toxicology, Medical University of Łódź, 90-752 Łódź, Poland; edward.kowalczyk@umed.lodz.pl; 6 Department of Allergology and Respiratory Rehabilitation, Medical University of Łódź, Żeligowskiego 7/9, 90-752 Łódź, Poland; monika.sienkiewicz@umed.lodz.pl

**Keywords:** melanin, carvacrol, agricultural residues, carboxymethyl cellulose, bioactive films, functional films, antioxidant activity, antimicrobial activity

## Abstract

Preparation of biodegradable packaging materials and valorisation of food industry residues to achieve “zero waste” goals is still a major challenge. Herein, biopolymer-based (carboxymethyl cellulose—CMC) bioactive films were prepared by the addition, alone or in combination, of carvacrol and fungal melanin isolated from champignon mushroom (*Agaricus bisporus*) agro-industrial residues. The mechanical, optical, thermal, water vapour, and UV-Vis barrier properties were studied. Fourier-transform infrared (FT-IR) spectroscopy studies were carried out to analyse the chemical composition of the resulting films. Antibacterial, antifungal, and antioxidant activities were also determined. Both CMC/melanin and CMC/melanin/carvacrol films showed some antimicrobial activity against *Escherichia coli*, *Staphylococcus aureus*, and *Candida albicans*. The addition of melanin increased the UV-blocking, mechanical, water vapour barrier, and antioxidant properties without substantially reducing the transparency of the films. The addition of carvacrol caused loss of transparency, however, composite CMC/melanin/carvacrol films showed excellent antioxidant activity and enhanced mechanical strength. The developed bioactive biopolymer films have a good potential to be green bioactive alternatives to plastic films in food packaging applications.

## 1. Introduction

The packaging industry is currently dominated by synthetic polymers (plastics) due to their low price and excellent functionality (mechanical strength and high barrier properties). Based on available data, the annual plastics production exceeds 400 million tons, and around 40% is used for packaging purposes [1]. Petroleum-based packaging materials are increasingly falling-out of favour due to their unsustainable production and the environmental burden of plastic [1,2]. The development of biodegradable packaging materials is an effective alternative to synthetic packaging materials based on petrochemical products [3,4]. Natural biopolymers and synthetic polymers based on annually renewable resources are the basis of a 21st century portfolio of sustainable, eco-efficient plastics. Biodegradable polymers have a great potential to be used as a green alternative to plastic packaging films as they become more and more affordable [3,5]. Biodegradable polymers from renewable resources have attracted a lot of academic and industrial attention worldwide. They are defined as polymers that undergo microbially-induced chain scission, leading to mineralization. Carbohydrates are used to manufacture biopolymer packaging films due to their excellent film-forming ability, good gas barrier properties, and mechanical properties [1]. Among naturally occurring biopolymers, cellulose is the most abundant one. Its derivatives have several advantages, such as recyclability, high viscosity, nontoxicity, biodegradability, and cost effectiveness [6,7,8,9,10,11]. Sodium carboxymethyl cellulose is a water-soluble cellulose derivative with carboxy methyl groups attached to some of hydroxyl groups of glucopyranose monomers of cellulose backbone. CMC has received scientific attention due to its polyelectrolyte character. In view of its high transparency, good film-forming property, large mechanical strength, non-toxicity, and biodegradability, it is found to be suitable for applications such as packaging material (for films and coatings), medicine, flocculating agent, chelating agent, emulsifier, thickening agent, water-retaining agent, and sizing agent [6,7,8,9,10,11].

In recent years, due to the current planet issues, the need for transition to a circular economy model based on the development of new strategies for making the best use of resources and for the elimination of the concept of wastes along the supply chain. In this model materials are recycled (a process in which wastes are transformed into value-added products by making them input elements for other products) and re-circulated during processing created a concept “waste = valuable resource” [12]. The food processing industries produce millions of tons of losses and waste during processing which is becoming a grave economic, environmental, and nutritional problem. These wastes can be a meaningful source of bioactive compounds [2,12,13,14]. Thus, these by-products can be exploited again in the food industry to develop functional ingredients and new foods or natural additives, or, in other industries, such as the pharmaceutical, agricultural, or chemical industries to obtain bioactive compounds [14].

Melanins is a common name of heterogenous group of dark-coloured biopigments, with high molecular weight. They are derived from the oxidation of monophenols and the subsequent polymerization of intermediate *o*-diphenols and their resulting quinones [15]. Melanins are known from their multifunctionality, including antioxidant, radioprotective, thermo-regulative, chemoprotective, antitumor, antiviral, antimicrobial, immunostimulating, and anti-inflammatory activities [13,16,17,18,19]. Recently, melanins have emerged as potential nanofillers and polymer matrix modifiers for food packaging polymers, such as: whey protein concentrate/isolate (WPI/WPC) [2], gelatine [4], poly(lactic acid) (PLA) [3], alginate [20], agar [21], carrageenan [22], cellulose [23], chitosan [24], poly(vinyl alcohol) [25], polypropylene/poly(butylene adipate-co-terephthalate) [26], polyhydroxybutyrate [27], and ethylene-vinyl acetate copolymer [28]. As with many other natural biopolymers, melanins can be obtained from renewable and natural resources. Moreover, they are non-toxic. Due to these characteristics, melanins have potential to be a “green” alternative to many existing commercial food additives. Furthermore, the possibility of melanins production by sustainable extraction from natural agricultural residues (e.g., waste from the harvesting of champignon mushroom, *Agaricus bisporus,* or residual watermelon seeds) has been demonstrated [13,17]. Another potential bioactive functional compound is carvacrol (CV, 5-isopropyl-2-methylphenol), a phenolic compound found primarily in oils of oregano, thyme, and marjoram, and recognized as a safe food additive (Generally Recognized as Safe—GRAS) [7,29,30,31,32,33,34,35,36]. This bioactive compound possesses antimicrobial properties, antioxidant, and a particular aroma which makes an attractive ingredient for certain types of foods [37,38]. Moreover, CV has been also reported to be used for modification of biopolymer [5,30,31,36,39,40,41,42,43,44], and synthethic films [45,46].

The functional properties of composite films are also essential in active and intelligent food packaging applications. A functional CMC-based film can be developed by adding functional materials such as bioactive compounds. The addition of bioactive compounds is expected to improve the film’s physical and functional properties. Considering the potential of melanins and carvacrol as functional materials, some synergies can be expected when these materials are used together. Moreover, the application of bioactive and biodegradable films seem to be an excellent alternative to reduce food loss and waste and to improve food security [5,41].

The main aim of this study was to investigate the effect of adding melanin obtained from *A. bisporus* waste, alone or in combination with carvacrol on the properties of carboxymethyl cellulose films (CMC). To the best of our knowledge, there is a lack of reporting about the modification of CMC with natural melanin and carvacrol to modify the functionality of composite biopolymer films. Fourier-transform infrared (FT-IR) spectroscopy was used to examine the chemical composition of films after active compounds addition. Moreover, we also examined the influence active agents on the colour and optical properties of the films. In order to evaluate the potential of resulted films as bioactive materials, their mechanical, barrier, antioxidant, and antimicrobial properties were determined.

## 2. Materials and Methods

### 2.1. Materials and Reagents

Sodium carboxymethyl cellulose (CMC) (degree of substitution = 0.7, M.W. = 90,000), carvacrol (natural, originated from thyme essential oil, 99%, food grade), calcium chloride, sodium chloride, hydrogen peroxide, disodium phosphate, monosodium phosphate, 2,2-diphenyl-1-picrylhydrazyl (DPPH), 2,2′-azino-bis(3-ethylbenzothiazoline-6-sulfonic acid) (ABTS), potassium persulphate, potassium ferricyanide, trichloroacetic acid, ferric chloride, iron sulphate, tris(hydroxymethyl)aminomethane, pyrogallol, resazurin, were purchased from Merck Chemical (Saint Louis, MO, USA). Tween 80, glycerol, ammonia water, hydrochloric acid, sodium hydroxide, chloroform, ethyl acetate, ethanol, and methanol were procured from Chempur (Piekary Śląskie, Poland). Mueller–Hinton broth, Mueller–Hinton agar, and agar-agar were purchased from Merck Chemical (Saint Louis, MO, USA). All chemicals were of analytical grade. *Escherichia coli* ATCC25922, *Staphylococcus aureus* ATCC43300, and *Candida albicans* ATCC10231 were purchased from ATCC (American Type Culture Collection, Manassas, VA, USA).

### 2.2. Isolation, Purification and Preparation of Melanin from Agaricus Bisporus Waste

Waste from the production of *A. bisporus* (Agaricus Bisporus Waste—ABW) in the form of stipes was obtained from a local producer in Wolsztyn (Wielkopolskie voivodeship, Poland). The isolation and purification of melanin was carried out as described elsewhere [13]. In brief, 500 g of ABW was first homogenised (Heidolph Brinkmann Homogenizer Silent Crusher, Schwabach, Germany) in 500 mL of distilled water and incubated (24 h, 37 °C) to allow acting of tyrosinase. After incubation the homogenate mixture was adjusted to pH = 10 by 1 M NaOH and incubated (24 h, 65 °C) to allow spontaneous polymerization of resulting *o*-diphenols and quinones to form melanin. Then, the mixture was filtered, centrifuged (6000 rpm, 10 min) and alkaline ABW raw melanin (ABW-RM) mixture was used to purify melanin. Alkaline ABW-RM mixture was first adjusted to pH 2.0 with 1 M HCl to precipitate melanin, followed by centrifugation at 6000 rpm for 10 min and a pellet was collected. The acid hydrolysis was then carried out (6 M HCl, 90 °C, 2 h). The resulted melanin was subsequently centrifuged (6000 rpm, 10 min) and washed by distilled water five times to neutral pH, then rinsed with organic solvents (chloroform, ethyl acetate and ethanol) three times to wash away lipids and other residues. Finally, the purified melanin was dried and ground to a fine powder in a mortar.

### 2.3. Determination of Carvacrol Minimum Inhibitory Concentration (MIC)

The minimal inhibitory concentration (MIC) (the lowest concentration of an antimicrobial that will inhibit the visible growth of a microorganism after overnight incubation) values of the carvacrol against bacteria (*Escherichia coli* ATCC25922 and *Staphylococcus aureus* ATCC43300), as well as yeast (*Candida albicans* ATCC10231), were determined by broth microdilution method according to Clinical and Laboratory Standards Institute (CLSI), with the following modification [29,47,48]: a final concentration of 1.0% (*v*/*v*) Tween 80 (filter sterilized) was incorporated into the medium to enhance oil solubility. We performed two-fold dilutions (1000–3.91 µg/mL); each well containing 50 µL of the tested carvacrol and 50 µL of bacterial or yeast suspension at a final concentration of 10^6^ CFU/mL (CFU–colony forming unit). Bacterial and yeast suspensions were prepared from 18 h cultures using saline. All tests were performed in duplicate. The MIC was estimated after 18 h of incubation at 37 °C in Mueller–Hinton broth (MHB) using resazurin. MIC was determined on the basis of the blue colour appearance in the first tested well after 3 h of incubation with resazurin. The colour change from blue to pink after 3 h of incubation with resazurin at 37 °C indicated the presence of viable microorganisms. To exclude an inhibitory effect of 1.0% Tween 80 on the microbial growth, the control assays with MHB and MHB supplemented with 1.0% Tween 80 were performed. Using the known concentrations of carvacrol, the final result was expressed in µg/mL.

### 2.4. Preparation of Films

In order to obtain CMC concentration of 2% (*w*/*w*) in the final film-forming solutions, CMC was weighed and completely dissolved in distilled water at 50 °C under continuous agitation (in tightly closed glass bottles). The pH of each solution was adjusted to 8.0 with ammonia water. Then, melanin was added to obtain concentrations of 0.1 and 0.5% (*w*/*w*). The mixtures were stirred (250 rpm) for 1 h at 50 °C to complete dissolve the melanin. After cooling, glycerol (at 5% (*w*/*w*), on a film-forming solution basis) and Tween 80 (0.5% *v*/*v*) were added and homogenized. The neat CMC films (without melanin addition) were prepared the same way, and served as reference materials. The film forming solutions were then divided into two batches. The samples only with melanin were described as CMC + 0.1 M and CMC + 0.5 M. CMC/melanin films with carvacrol were prepared using an emulsion method. To one batch of CMC/melanin film forming solutions 0.025% (*w*/*v*–250 µg/mL) of carvacrol (CV) was then added and stirred (250 rpm) until a homogenous appearance of solution was obtained (approximately 30 min) and the samples were described as CMC + 0.1 M + CV and CMC + 0.5 M + CV. CMC films with carvacrol (CMC + CV) were also produced following the same procedure. All CMC-based film variants were prepared in 10 repetitions. The film-forming solutions were cast on square (120 × 120 mm) polystyrene plates and dried at 40 °C for 48 h. The dried films were peeled off from the plates and were conditioned at 25 °C and 50% RH (relative humidity) in a temperature and humidity clean room prior to any tests [2].

### 2.5. Thickness, Moisture Content (MC) and Mechanical Properties of The Films

To determine MC of the films, the samples were dried at 105 °C for 24 h, and the weight change was analysed [2]. The thickness of all samples was measured with a hand-held micrometer (Dial Thickness Gauge 7301, Mitoyuto Corporation, Kangagawa, Japan) with an accuracy of 0.001 mm. Each film was measured in five random points and the results were averaged. The mechanical properties of the samples were determined with Zwick/Roell 2.5 Z equipment (Ulm, Germany). Static tensile testing was carried out to assess tensile strength and elongation at break (the gap between tensile clamps was 25 mm and crosshead speed was 100 mm/min).

### 2.6. DSC Measurements

A differential scanning calorimetry (DSC) calorimeter (DSC 3 Star System, Mettler-Toledo LLC, Columbus, OH, USA) was used to determine thermal properties of the samples. The tests were carried out over a temperature range from 10 to 300 at φ = 10°/min and under nitrogen flow (50 mL/min), performing two heating and one cooling scans according to PN-EN ISO 11357-03:2018-06 norm [2].

### 2.7. The Water Vapour Transmission Rate (WVTR) of the Films

Water vapour transmission rate (WVTR) was performed according to DIN 53122-1 and ISO 2528:1995 norms as described elsewhere [49]. WVTR was measured by means of a gravimetric method that is based on the sorption of humidity by CaCl_2_ and a comparison of sample weight gain. Initially, the amount of dry CaCl_2_ inside the container was 9 g. The area of film samples was 8.86 cm^2^. Measurement was carried out for a period of 4 days and each day the containers were weighed to determine the amount of absorbed water vapour through the films. The results were expressed as average values from each day of measurement and each container. Analyses were carried out at 10 independent containers (10 repetitions) for each type films, calculated as a standard unit g/(m^2^ × Day) and presented as a mean ± standard deviation.

### 2.8. Spectral Analysis

The UV–Vis blocking properties of the film samples were determined using a UV–Vis Thermo Scientific Evolution 220 spectrophotometer (Waltham, MA, USA). The tests were carried out in a range of 300 to 700 nm, by putting particular samples directly on quartz cuvette (Bionovo, Legnica, Poland). FT-IR spectroscopy was used in order to assess the chemical composition of obtained films, as described previously. Firstly, a 4 cm^2^ squares of each film were cut from the samples, then were analysed directly on the ray-exposing stage of the ATR (Attenuated Total Reflectance) accessory of a Perkin Elmer Spectrum 100 FT-IR spectrometer (Waltham, MA, USA) operating in ATR mode. Spectra (64 scans) were recorded over a wavenumber range of 650 to 4000 cm^−1^ at a resolution of 4 cm^−1^ [2]. For analysis, all spectra were baseline corrected and normalized using SPECTRUM^TM^ software v10.

### 2.9. Colour Analysis

The effect of melanin on the colour of the films was measured using a colorimeter (CR-5, Konica Minolta, Tokyo, Japan). The values measured were L* (white 100 and black 0), a* values (red positive and green negative), and b* values (yellow positive and blue negative). For each film type five samples were analyzed by taking three measurements on both sides of each sample. The Whiteness Index (WI), Yellowness Index (YI), total colour difference (ΔE), chroma (C), and hue angle (H°) were calculated according the following formulas (1–5) [2,4]:

(1) $$\mathrm{WI}=100-{\left[\left(100-L^{*}\right)+a^{2}+b^{2}\right]}^{0.5}\;\;\;$$

(2) $$\mathrm{YI}=142.86\times b\times L^{-1}\;\;\;\;\;$$

(3) $$\Delta E={\left[{\left(L_{\mathrm{standard}\;}-L_{\mathrm{sample}}\right)}^{2\;\;}+\;{\left(a_{\mathrm{standard}}-a_{\mathrm{sample}}\right)}^{2}\;+{\left(b_{\mathrm{standard}}-b_{\mathrm{sample}}\right)}^{2}\right]}^{0.5}\;$$

(4) $$C=\mathrm{arctg}\frac{b_{\mathrm{sample}}}{a_{\mathrm{sample}}}\;\;\;\;\;$$

(5) $$H{}^{\circ}\;={\left[{\left(a_{\mathrm{sample}}\right)}^{2}+{\left(b_{\mathrm{sample}}\right)}^{2}\right]}^{0.5}\;$$

### 2.10. Antioxidant Activity and Reducing Power

The reducing power, DPPH, ABTS^+·^, and O_2_^−^ radicals scavenging activities were analysed based on methodologies described in our previous study [2]. The reducing power was determined by placing the film samples (100 mg) in 1.25 mL of phosphate buffer (0.2 M, pH 6.6) followed by the addition of 1.25 mL of 1% potassium ferricyanide solution. Samples were then incubated for 20 min at 50 °C followed by the addition of 1.25 mL of trichloroacetic acid. Subsequently, the test tubes were centrifuged at 3000 rpm for 10 min. Then, 1.25 mL of obtained supernatant was diluted with 1.25 mL of deionized water. Finally, 0.25 mL of 0.1% ferric chloride solution was added and the absorbance was measured at 700 nm [2].

To determine DPPH radical scavenging activity 100 mg of each film was placed in 25 mL of 0.01 mM DPPH methanolic solution, incubated for 30 min at room temperature and absorbance at 517 nm was measured. As a control, the same solution was measured, but without any film samples. In total, 10 mL of ABTS^+^ solution was mixed with 100 mg of the films and the absorbance was measured at 734 nm. To determine O_2_^−^ radical scavenging activity, 3 mL of 50 mmol/L (pH 8.2) Tris-HCl buffer was mixed with 100 mg of the films. Then, a pyrogallol solution (0.3 mL, 7 mmol/L, preheated to 25 °C) was added, and allowed to react for exactly 4 min. Finally, 1 mL od 10 mmol/L of HCl was added to terminate the reaction and absorbance was measured at 318 nm [2].

### 2.11. Antimicrobial Activity

The film samples were cut into square shapes (3 cm × 3 cm) and their antimicrobial properties were carried out according to the ASTM E 2180-01 standard with modification described elsewhere [50]. As the first step of the experiments, *E. coli*, *S. aureus*, and *C. albicans* cultures originated from 24 h growth (coming from stock cultures) were prepared. The concentrations of the cultures were standardized to 1.5 × 10^8^ CFU/mL. The concentration of each culture was measured using Cell Density Meter (WPA-CB4, Cambridge, UK). The agar slurry was prepared by dissolving 0.85 g of NaCl and 0.3 g of agar–agar in 100 mL of deionized water and autoclaved for 15 min at 121 °C and equilibrated at 45 °C (one agar slurry was prepared for each strain). Then, 1 mL of the culture (separately) was placed into the 100 mL of agar slurry. The final concentration of each culture was 1.5 × 10^6^ CFU/mL in molten agar slurry. The square samples of each film were introduced (separately) into the sterile Petri dishes with a diameter of 55 mm. Inoculated agar slurry (1.0 mL) was pipetted onto each square sample. The samples were incubated 24 h at 30 °C with relative humidity at 90%. After incubation the samples were aseptically removed from the Petri dishes and introduced into the 100 mL of MHB. The samples were dispersed 1 min in the Bag Mixer^®^ CC (Interscience, St Nom la Brètech, France). The dispersion facilitated the complete release of the agar slurry from the samples. Then serial dilutions of the initial inoculum were performed. Each dilution was spread into the Mueller–Hinton Agar and incubated at 30 °C for 48 h. The results were presented as an average value with standard deviations.

### 2.12. Statistical Analysis

For statistical analysis of the obtained results Statistica software version 10 was used (StatSoft Polska, Kraków, Poland). Differences between means were determined using analysis of variance (ANOVA), followed by Fisher’s LSD (Least Significant Difference) post-hoc. The significance of each mean property value was determined (*p* < 0.05). All measurements were carried out in at least three repetitions.

## 3. Results and Discussion

### 3.1. Antimicrobial Activity of The Films

**Figure 1:**
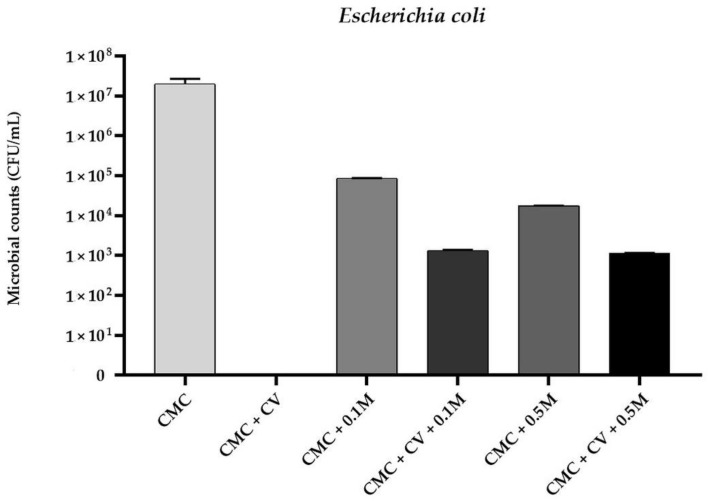
The effect of CMC-based neat and modified films on viability of *Escherichia coli* cells.

**Figure 2:**
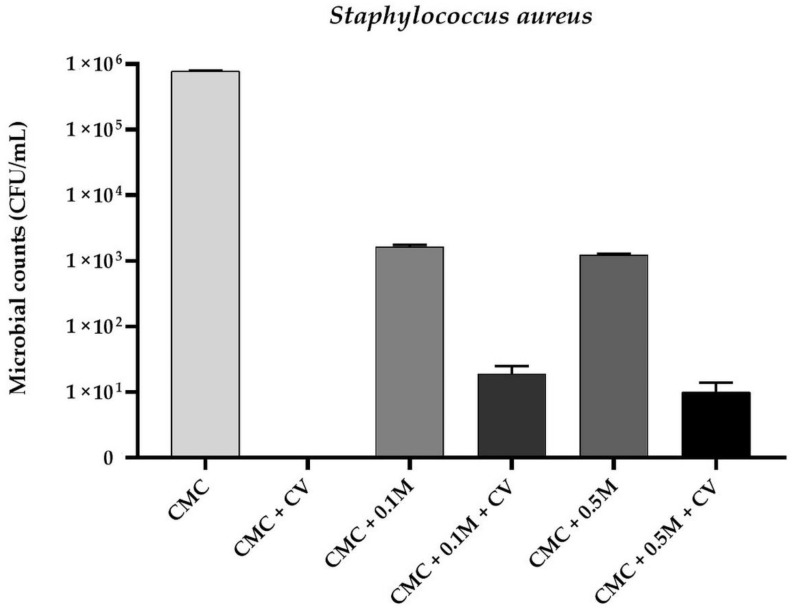
The effect of CMC-based neat and modified films on viability of *Staphylococcus aureus* cells.

**Figure 3:**
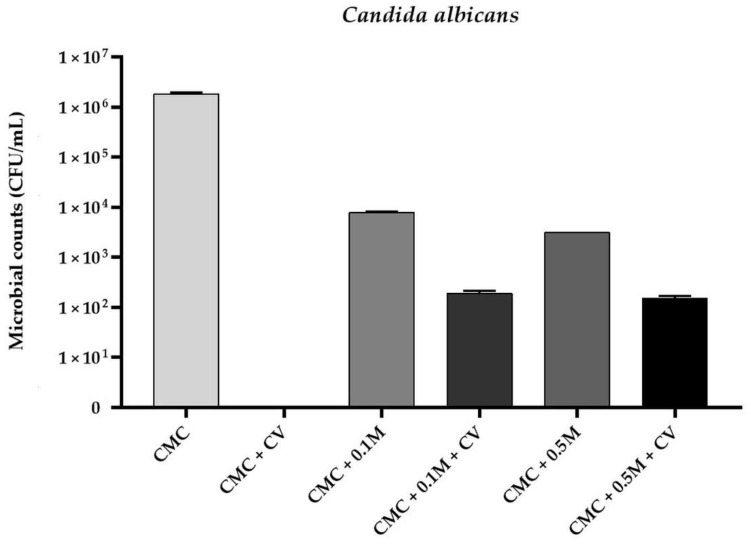
The effect of CMC-based neat and modified films on viability of *Candida albicans* cells.

Based of MIC determination results it was observed that the MIC values of CV for *E. coli*, *S. aureus*, and *C. albicans* were 256 µg/mL, 128 µg/mL, and 256 µg/mL, respectively. A comparable finding was reported by other authors [32,33,34]. Therefore, CV was added to CMC filmogenic solutions in concentration of 0.025% (*w/v*–250 µg/mL). The antimicrobial activity of CMC-based films is presented in Figure 1, Figure 2 and Figure 3. No antimicrobial activity was noticed for neat CMC film. A complete reduction in microbial counts was observed for CMC + CV samples (*p* < 0.05). Moreover, reduction in bacterial and fungal counts was observed for CMC/melanin samples (*p* < 0.05). For sample CMC + 0.5 M the level of *E. coli* was 1.14 × 10^4^ ± 0.13 CFU/mL, for *S. aureus* 1.03 × 10^3^ ± 0.22 CFU/mL, whereas for *C. albicans*, 3.66 × 10^3^ ± 0.27 CFU/mL was noticed. Interestingly, a significant reduction was also observed in case of CMC + 0.1 M + CV and CMC + 0.5 M + CV samples, however, not complete, as noticed for sample CMC + CV. The observed results suggest that this effect can be presumably attributed to some interactions between melanin and carvacrol in CMC matrix, lowering CV diffusion efficiency. The antibacterial activity of chitosan/CV [51], chitosan/CV/pomegranate peel extract [40], polypropylene/CV [31], flaxseed gum/CV [36,44], and PLA/CV [52] films, as well as PLA/CV [53] nanofibers, has been already reported. Carvacrol is a phenolic compound with a hydroxyl group on an aromatic ring. The hydroxyl group of CV plays a crucial role in the antibacterial activity of this phytochemical [32,33,34]. Indeed, CV interacts with the lipid bilayer of the bacterial cytoplasmic membrane due to its hydrophobic nature and aligns itself between fatty acid chains causing the expansion and destabilization of the membrane structure by increasing its fluidity and permeability for protons and ions (mainly H^+^ and K^+^) [7,35,36,44]. The loss of the ion gradient leads to bacterial cell death [36,44]. However, the mechanisms of action of CV against *Candida* have been investigated in several studies and seems to exert its antifungal activity by inducing envelope, disrupting membrane integrity, and blocking ergosterol biosynthesis [33]. Fungicidal activity of starch/CV [43], polyethylene/CV [46], and polypropylene/CV [45] films, as well as polyvinyl alcohol/CV coatings [42] was reported. The literature on melanin applications to develop antimicrobial properties of polymer film is relatively limited. It is suggested that melanin antibacterial activity might result from damage of the cell membrane and affect bacteria membrane function [16]. In previous study PLA/melanin film showed antimicrobial effect against some food-borne pathogenic bacteria [3]. Kiran et al. synthetized nanomelanin-polyhydroxybutyrate nanocomposite film which showed a strong protective effect against multidrug-resistant *S. aureus* [27].

### 3.2. Radicals Scavenging Activities and Reducing Power

The antioxidant capacity of the films was determined as the reducing power and radicals (DPPH, ABTS, O_2_^−^) scavenging activity and the results are summarized in Table 1. The CMC control film showed low reducing power (0.030 ± 0.001) and radical scavenging activities (15.23 ± 0.01%, 7.98 ± 0.14%, and 9.68 ± 0.05%, for DPPH, ABTS, and O_2_^−^, respectively) which is consistent with reports of other authors [6,8]. A significant increase in reducing power and radical scavenging activities was noticed when melanin was added (*p* < 0.05). Furthermore, a dose-dependent increment of antioxidant activity was noticed. The antioxidant activity of melanin-modified films has been already observed in numerous studies using melanin in a form of fillers [3,21,22,23], as well as when dissolved in a film-forming alkaline solutions [2,4]. The antioxidant activity of melanin from *A. bisporus* was previously reported [13]. In general, melanins act as very effective antioxidants due to presence of intramolecular non-covalent electrons, having ability to easily interact with free radicals and other reactive species [23]. It was also observed that CMC + CV sample showed significantly higher reducing power (0.192 ± 0.002) and antioxidant activity (45.44 ± 0.02%, 69.18 ± 0.05%, and 61.63 ± 0.09%, for DPPH, ABTS, and O_2_^−^, respectively) when compared with neat CMC (*p* < 0.05). The antioxidant capacity of carvacrol depends on the steric and electronic effect of its ring, besides the presence of the hydroxyl group which is capable of donating hydrogen atoms [7,44,51,52]. This findings are consistent with results of other authors, reporting antioxidant activities of chitosan/CV [51], polypropylene/CV [31], poly(lactic acid)/poly(ε-caprolactone)/CV [52], and flaxseed gum/CV films [36].

It is worth noting that CMC + 0.1 M + C and CMC + 0.5 M + C samples showed significantly higher antioxidant activity than films only with melanin (*p* < 0.05) with the highest activity of sample CMC + 0.5 M + C (72.70 ± 0.05%, 93.54 ± 0.03%, and 89.84 ± 0.07%, for DPPH, ABTS, and O_2_^−^, respectively). Those results are higher than reported for WPI/WPC/melanin [2], as well as flaxseed gum/CV films [36], but comparable with results reported for whey protein isolate nanofibrils–carvacrol films [54]. This effect might be attributed to synergistic effect of both antioxidants present in polymer matrix [40,46]. In fact, it is already established that the antioxidant activity of polymer-based films is directly dependent on the content of antioxidant compounds present in composite materials [2,23,40,46]. A similar synergistic effect of CV with other antioxidants such as pomegranate peel extract [40], whey protein isolate nanofibrils [54], as well as in inclusion complexes with cinnamaldehyde [46] was reported. The hight antioxidant activity of CMC-modified films shown their potential to be used in active antioxidant packaging to prevent oxidation-sensitive food matrices, as well as to increase their shelf life. In fact, the preservative activity against pork lard rancidity of gelatine-based coatings modified with fungal was reported [55]. Similar effect was showed by Wang et al. who applied whey protein isolate nanofibrils-based films with CV on fresh-cut cheese [54]. CV-incorporated flaxseed gum-sodium alginate films reduced formation of total volatile base nitrogen (TVB-N) resulted from activity of aerobic spoilage microflora [44].

### 3.3. The Thickness, Moisture Content, Mechanical, Thermal and Water Vapour Barrier Properties of The Films

The thickness, TSC (Total Solids Content), mechanical, thermal, and water vapour barrier properties are listed in Table 2. The lowest TSC was noticed for neat CMC film (89.00 ± 0.31%). The addition of melanin, as well as carvacrol caused the increase in TSC of the films (*p* < 0.05), which is consistent with results reported in other studies, where melanins, as well as carvacrol, were used to modify biopolymers films [2,21,52]. In contrast, Dhumal et al. found that the impregnation of carvacrol did not influence the moisture content in starch–guar gum films [30]. In terms of thickness, only CMC + CV and CMC + 0.1 M + CV films showed significantly higher values (0.12 ± 0.02 mm and 0.08 ± 0.00 mm, respectively). In the previously reported study, the thickness of WPI/WPC films with the same melanin concentrations was not affected, because of a small amount of melanin used [2]. Hence, as the films only with melanin addition did not show significant increase in thickness, probably the increase in CMC + CV and CMC + 0.1 M+CV samples was resulted by interactions of CV and CMC (caused tighter binding of film matrix), not by higher solids content [8,9].

It was noticed that tensile strength of CMC + 0.5 M (25.89 ± 3.06 N) as well as CMC-CV (25.30 ± 1.52 N) films was enhanced in comparison with neat CMC film (18.11 ± 1.25 N) (*p* < 0.05). The improvement of mechanical strength of melanin-modified films has been already reported [2,4]. It should be pointed out, that the mechanical properties of CMC/melanin/CV film was significantly higher than corresponding CMC/melanin films, and can be attributed to synergistic interaction of film constituents. The mechanical properties of films are dependent on the microstructure of the film network, CMC composition, and intermolecular forces. The increase in film mechanical properties might be due to the intermolecular interaction of carboxyl group of CMC and hydroxyl group of melanin and CV molecules [8]. A decrease in the modified films EB in comparison to the control samples was observed (*p* < 0.05). This presumably resulted from strong hydrogen bonding (H-bonding) interactions between melanin, carvacrol, and the polymer matrix, which has been already reported to improve the mechanical properties of the films [2,21,23,24]. However, this results in contrary to results obtained for synthetic films from polypropylene and low-density polyethylene, as well as for chitosan-based films [40], where carvacrol was reported to increase EB due to its plasticizing effect [31,45].

Packaging films must be exposed to environment, hence adsorbtion and penetration of the water vapour is an important factor that controls the final product quality [36]. For better packaging films, low WVTR is required [23]. Although CV is reported to be hydrophobic [35,36,41], no significant WVTR differences of neat CMC (1098.68 ± 6.74 (g/(m^2^ × Day)) and CMC + CV (1092.38 ± 11.73 g/(m^2^ × Day)) were observed (*p* > 0.05), presumably linked with small amount of carvacrol used. Similarly, Medina-Jaramillo et al. reported that the low concentrations of CV added (0.03%, 0.06%, and 0.09%) did not cause changes in the water vapour barrier properties of the alginate coatings [41]. In contrast, Du et al. found that the addition of carvacrol (0.5%, 1.0%, and 1.5%) to tomato puree films increased WVTR compared to the control [39], similarly for chitosan-based films modified with 0.5% and 1.0% of CV as reported by Flores et al. [5]. However, Dhumal et al. who used carvacrol to modify starch–guar gum films, stated that the incorporation of essential oils compounds may reduce the impact of plasticizers within the biopolymer matrix, thereby lowering the moisture transmission rates [30]. On the other hand, a significant decrease in WVTR was observed when melanin was added (*p* < 0.05). Reduction in the WVTR with increasing melanin content results in an improvement of the functional properties of these films, considering the hydrophilic characteristics of the matrix. The lowest WVTR was observed for sample CMC + 0.5 M (933.07 ± 4.32 g/(m^2^ × Day)). Moreover, films only with melanin showed significantly lower WVTR than the corresponding films with melanin and carvacrol (*p* < 0.05). The observed WVTR decrease is consistent with previously reported results for WPC/WPI [2], alginate/poly(vinyl alcohol) [20], cellulose [23], as well as gelatine [4] films modified with various melanins, and could be attributed to highly hydrophobic properties of *A. bisporus* melanin [13]. Furthermore, in the film forming solution the polymeric chains may partially be immobilized at the interface with melanin. Consequently, the polymeric chains become less mobile, reducing the diffusibility of water via the CMC chains interface and leading to a decrease in WVTR. Therefore, the lower WVTR values obtained with melanin addition can be explained by formation of an interconnecting melanin network within the film matrix. The results for CMC modified film are lower than results reported for WPC/WPI films modified with melanin from watermelon seeds [2], but higher than reported for PLA films modified with A. bisporus melanin [3]. It was already reported that the effect of melanin on WVTR is concentration dependent. For agar–melanin nanoparticles composite films the incorporation of low melanin-particle content did not affect their WVTR, whereas at higher content, the WVTR increased [21]. When melanin was added to PLA composite films at low content, it was observed that WVTR increased, but decreased at high melanin content [3]. Regarding the thermal properties of the films it was observed that CV significantly improved melting temperature from 130.54 °C (CMC) to 144.93 °C (CMC + CV). A similar observation was reported for flaxseed gum/CV films [36]. Surprisingly, T_m_ of melanin-modified and melanin/CV-CMC films was lower than of neat CMC films. This result is in contrary to results obtained in previous study, where the addition of melanin increased the melting temperature of WPC/WPI films [2]. On the other hand it was reported that the addition of melanins as a fillers did not change melting temperatures of PLA [3], cellulose [23], and agar [21] films. However, a degradation of all the CMC-based samples was noticed when temperature exceeded 250 °C.

### 3.4. Appearance, Colour, Opacity, and Transparency Changes

**Figure 4:**
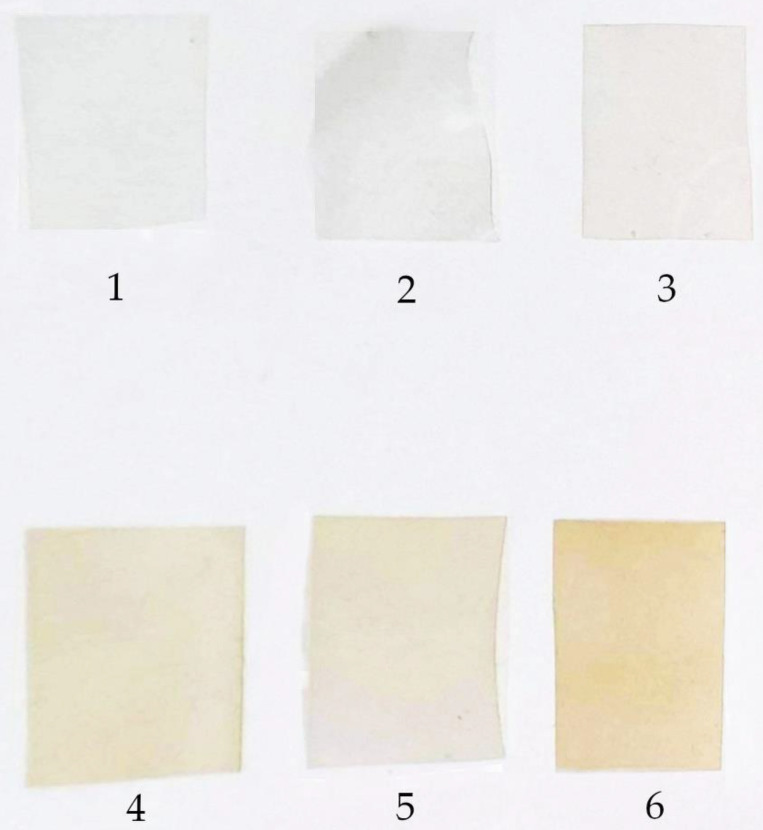
The visual appearance of CMC-based films. **1**–CMC; **2**–CMC + CV; **3**–CMC + 0.1 M; **4**–CMC + 0.5 M; **5**–CMC + 0.1 M +CV; **6**–CMC + 0.5 M + CV.

The colour coordinates, the total colour difference (ΔE), the yellowness index (YI), the whiteness index (WI), opacity, and transparency of the films are presented in Table 3, whereas the appearance of the films is illustrated in Figure 4. The neat CMC film was characterized by a high transparency (T_660_ = 82.07%) and was almost colourless. It was noticed that addition of carvacrol did not affect the L*, b*, YI, WI, and C parameters (*p* > 0.05), however a* and H°, increased due to the red–orange colour of pure carvacrol (*p* < 0.05). Moreover CMC-C film was less transparent (T660 = 79.10%) and had lower opacity (7.22 ± 0.13) than the neat CMC film (7.62 ± 0.20). The lowering of b* and WI values with the addition of CV was reported for apple puree/pectin/CV films [39], and chitosan/CV [51] films. Generally, films with CV were characterized by low transparency and were not see-through. As can be seen the addition of melanin significantly reduced lightness of the films (*p* < 0.05). Simultaneously, the a* and b* colour coordinates, as well as C and H° values of the modified films increased due to the red-brown colour of melanin (*p* < 0.05) [13]. The increased melanin concentration caused a significant increase in YI of the films (*p* < 0.05), whereas a decrease in WI was noticed (*p* < 0.05). The yellow and brown colour of melanin modified films was also reported in other studies [2,3,4,21,24]. Moreover, the opacity of the films decreased significantly when melanin concentration increased (*p* < 0.05). A similar effect was reported for PLA and gelatine films modified with *A. bisporus* melanin [3], as well as for chitosan-based coatings modified with CV [5]. Regarding the total colour difference (ΔE) of the films a significant increase was noticed (*p* < 0.05), and ranged from 1.13 ± 0.68 (CMC + C) to 14.24 ± 0.72 (CMC + 0.5 M + C). When ΔE is higher than 1.00, the human eye is able to percept the colour difference, thus modification with carvacrol, as well as melanin caused noticeable colour changes [2,3].

### 3.5. The Changes of UV-Vis Blocking Properties

**Figure 5:**
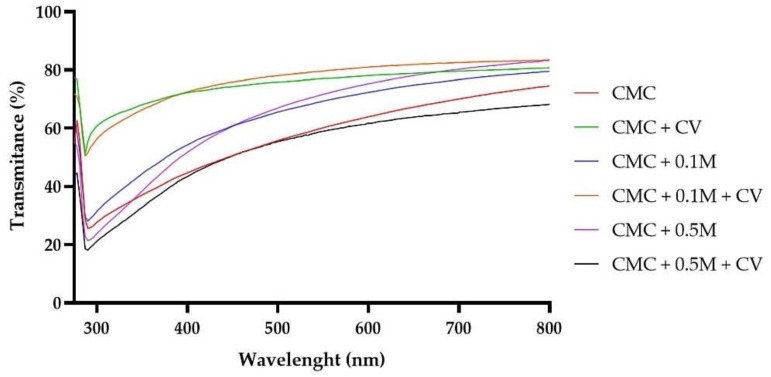
UV-Vis spectra of CMC-based neat and modified films.

Figure 5 presents the UV-Vis transmittance spectra of the neat and modified CMC films. The neat CMC film showed high transmittance in the range of 200 to 700 nm, indicating that the film is highly transparent to UV and visible light which also is in line with results presented in Table 3, and reports of other authors [6]. Although CMC + C film showed lower opacity, displayed moderate transparency to visible light and no UV-blocking effect. A concentration-dependent decrease in CMC films transmittance was noticed, which indicates that melanin, even at low concentration (0.1%), improved UV–Vis blocking effect of the films. This effect can be attributed to the absorption of UV light by melanin and was reported also for gelatine/melanin [4], as well as WPC/WPI/melanin films [2]. It should be pointed out that one of the main functions of melanins in nature is protection against UV radiation, due to their strong UV-absorbing properties [15]. It was already reported, that melanin from A. bisporus has strong UV–Vis barrier properties [13]. Interestingly, samples CMC + 0.1 M + C and CMC + 0.5 M + C displayed lower transparency than the corresponding CMC + 0.1 M and CMC + 0.5 M films. As mentioned, samples with carvacrol were opaque and the observed higher light-blocking properties seem to be synergistic effect of the absorption of UV light by melanin and light scattering by carvacrol droplets in polymer matrix. A blocking and scattering of the light path by melanins when used as nanofillers, was reported for PLA/melanin [3], and agar/melanin films [21]. High UV-blocking properties of modified films might help to protect packaged food from oxidative deterioration caused by UV radiation which leads to discoloration, nutrient loss and off-flavour production.

### 3.6. FT-IR Results

**Figure 6:**
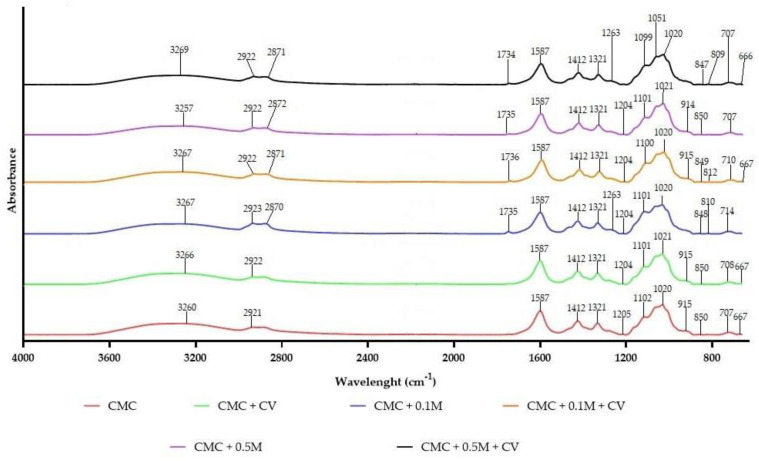
Fourier-transform infrared (FT-IR) spectra of CMC-based neat and modified films.

FT-IR is a valuable technique used to determine the miscibility and compatibility of polymeric matrices and additives, due to its rapid and non-destructive nature [23]. Figure 6 presents FT-IR spectra of CMC-based films. A broad absorption band at about 3200–3350 cm^−1^ in the CMC films was due to the stretching of hydroxyl groups of cellulose, melanin and carvacrol [2,13,36,52,56,57]. Stretching peaks at approximately 2920 cm^−1^ (CH_3_) and 2870 cm^−1^ (CH_2_) were also detected [36]. The two absorption peaks at 1587 and 1412 cm^−1^ were attributed to the asymmetric and symmetric stretching vibration of carboxylic groups, respectively. They correspond also to the vibration of melanin ans carvacrol aromatic C=C bonds [2,13,52]. There were two more bands at 1100 and 1020 cm^−1^ related to the stretching of C–O in polysaccharide. The peak at 1320 cm^−1^ was due to the O–C–H and H–C–H deformation and absorption bands at 1260 and 900 cm^−1^ were related to the C–H vibrations of CMC and plasticizer (glycerol) [2,6,8,57]. Generally, no substantial variations were noted in the functional groups of CMC modified films. The obtained results suggest that there were no structural changes in CMC films due to the addition of melanin and carvacrol. The physical interactions (H–bonding, van der Waals force) between CMC-melanin-carvacrol are presumably the cause of minor shifts and small changes in intensities, which in agreement with other reports [2].

## 4. Conclusions

The properties of modified CMC-based films incorporated with fungal melanin and carvacrol (alone or in combination) were explored in this study. The properties of CMC-composites were compared to neat CMC film. Melanin played a vital role in mechanical, antioxidant, antimicrobial, and barrier properties. The increase in tensile strength and water vapour barrier properties as well as decrease in elongation at break were observed for CMC/melanin and CMC/melanin/CV films. CMC/CV films caused the total reduction in viable bacteria and yeast, however, CMC/melanin/CV films also showed good antimicrobial activity. All modified films showed antioxidant activity and the highest level (72.70–93.54% of radicals scavenging activities) was observed for sample CMC + 0.5 M + CV which was attributed to synergistic action of both active compounds. An improvement in the antioxidant and antimicrobial activities of modified CMC films is worth mentioning as an important aspect of the work. Considerable improvement in these properties has been observed in comparison to pure CMC. The results described here are particularly interesting if one considers that the additives used have a natural origin, providing added value in the development of sustainable alternatives to traditional synthetic antioxidants. We conclude that the obtained bioactive CMC-modified films could be potentially used for active food packaging applications. However, further tests of the influence of the developed materials (in the form of films and coatings) on various food products should be carried out to determine their suitability in food technology.

## Figures and Tables

**Table 1:** Reducing power (RP) and radicals scavenging activity of CMC-based films.

Sample	RP (700 nm)	DPPH (%)	ABTS (%)	O_2_^−^ (%)
CMC	0.030 ± 0.001 ^f^	15.23 ± 0.01 ^f^	7.98 ± 0.14 ^f^	9.68 ± 0.05 ^f^
CMC + 0.1 M	0.206 ± 0.005 ^d^	64.20 ± 0.06 ^d^	55.72 ± 0.08 ^e^	63.92 ± 0.07 ^d^
CMC + 0.5 M	0.244 ± 0.010 ^c^	65.73 ± 0.01 ^c^	57.65 ± 0.08 ^d^	68.08 ± 0.09 ^c^
CMC + CV	0.192 ± 0.002 ^e^	45.44 ± 0.02 ^e^	69.18 ± 0.05 ^c^	61.63 ± 0.09 ^e^
CMC + 0.1 M + CV	0.259 ± 0.001 ^b^	69.70 ± 0.06 ^b^	77.61 ± 0.01 ^b^	81.83 ± 0.18 ^b^
CMC + 0.5 M + CV	0.389 ± 0.006 ^a^	72.70 ± 0.05 ^a^	93.54 ± 0.03 ^a^	89.84 ± 0.07 ^a^

Values are means ± standard deviation of triplicate determinations. Means with different letters in the same column are significantly different at *p* < 0.05.

**Table 2:** Thickness, total solids content (TSC), water vapour transmission ratio (WVTR), tensile strength (TS), elongation at break (EB), melting temperature (T_m_), and melting enthalpy (ΔH_m_) of CMC-based films.

Sample	Thickness (mm)	TSC (%)	WVTR (g/(m^2^ × Day))	TS (N)	EB (%)	T_m_ (°C)	ΔH_m_ (J/g)
CMC	0.05 ± 0.00 ^c^	89.00 ± 0.31 ^b^	1098.68 ± 6.74 ^a^	18.11 ± 1.25 ^d^	9.91 ± 0.25 ^a^	130.54	−252.30
CMC + 0.1 M	0.06 ± 0.01 ^c^	89.33 ± 0.28 ^ab^	962.42 ± 3.51 ^c^	19.61 ± 1.03 ^d^	8.74 ± 0.76 ^b^	105.51	−250.33
CMC + 0.5 M	0.04 ± 0.01 ^c^	89.35 ± 0.12 ^ab^	933.07 ± 4.32 ^d^	25.89 ± 3.06 ^c^	7.31 ± 0.66 ^cd^	113.04	−283.25
CMC + CV	0.12 ± 0.02 ^a^	89.02 ± 0.17 ^b^	1092.38 ± 11.73 ^ab^	25.30 ± 1.52 ^bc^	7.88 ± 0.31 ^bc^	144.93	−153.91
CMC + 0.1 M + CV	0.08 ± 0.00 ^b^	89.43 ± 0.19 ^ab^	992.03 ± 3.84 ^b^	29.20 ± 2.44 ^ab^	6.33 ± 0.49 ^d^	102.69	−201.18
CMC + 0.5 M + CV	0.05 ± 0.01 ^c^	89.57 ± 0.03 ^a^	961.74 ± 9.13 ^bc^	34.29 ± 1.83 ^a^	4.11 ± 0.72 ^e^	107.72	−288.62

Values are means ± standard deviation of triplicate determinations. Means with different letters in the same column are significantly different at *p* < 0.05.

**Table 3:** Colour, total colour difference (ΔE), chroma (C), hue angle (H^°^), yellowness index (YI), whiteness index (WI), opacity, and transmittance at 660 nm of CMC-based films.

Sample	L*	a*	b*	ΔE	C	H°	YI	WI	Opacity	T_660_ (%)
CMC	89.77 ± 0.92 ^a^	−0.66 ± 0.02 ^c^	4.38 ± 0.38 ^d^	used as standard	4.43 ± 0.38 ^c^	−8.68 ± 0.83 ^d^	6.97 ± 0.68 ^e^	94.54 ± 0.39 ^a^	7.62 ± 0.20 ^a^	82.07 ^a^
CMC + 0.1 M	86.08 ± 0.61 ^ab^	−0.31 ± 0.09 ^c^	6.75 ± 0.93 ^c^	2.78 ± 0.82 ^bc^	6.96 ± 0.94 ^b^	−2.71 ± 1.07 ^c^	10.98 ± 1.54 ^c^	92.84 ± 1.25 ^c^	7.50 ± 0.17 ^a^	78.72 ^c^
CMC + 0.5 M	83.81 ± 1.44 ^bc^	0.88 ± 0.03 ^b^	16.61 ± 0.38 ^b^	13.76 ± 0.64 ^a^	17.47 ± 1.40 ^a^	3.17 ± 0.64 ^a^	28.38 ± 4.47 ^a^	82.88 ± 2.37 ^d^	7.02 ± 0.13 ^b^	67.97 ^d^
CMC + CV	89.28 ± 0.50 ^a^	−0.08 ± 0.13 ^c^	4.94 ± 0.50 ^d^	1.13 ± 0.68 ^c^	5.04 ± 0.43 ^bc^	−0.56 ± 1.13 ^b^	7.91 ± 0.84 ^de^	94.07 ± 0.46 ^a^	7.22 ± 0.13 ^b^	79.10 ^b^
CMC + 0.1 M + CV	88.62 ± 0.22 ^ab^	−0.19 ± 0.09 ^c^	6.32 ± 0.93 ^cd^	3.62 ± 0.78 ^b^	6.06 ± 0.75 ^bc^	−1.91 ± 0.83 ^bc^	10.20 ± 1.51 ^c^	92.82 ± 0.83 ^b^	7.14 ± 0.15 ^b^	75.29 ^e^
CMC + 0.5 M + CV	82.50 ± 0.28 ^c^	1.35 ± 0.84 ^a^	19.85 ± 0.28 ^a^	14.24 ± 0.72 ^a^	17.82 ± 3.02 ^a^	4.26 ± 1.49 ^a^	20.13 ± 0.13 ^b^	86.64 ± 0.96 ^d^	6.54 ± 0.17 ^c^	64.37 ^f^

Values are means ± standard deviation of triplicate determinations. Means with different letters in the same column are significantly different at *p* < 0.05.

## Data Availability

The data presented in this study are available on request from the corresponding authors.
